# Supplementary material for: Is there ‘trustworthy’ evidence for using manual therapy to treat patients with shoulder dysfunction?: A systematic review
Source: PLoS One. 2024 Jan 18;19(1):e0297234. doi: 10.1371/journal.pone.0297234 (PMC10796022; doi:10.1371/journal.pone.0297234)
Supplement: S1 Appendix — (PDF) [file pone.0297234.s001.pdf]

S1 Appendix. Search Strategy

PUBMED

(randomized AND (control OR clinical)) AND shoulder pain OR shoulder impingement syndrome OR adhesive capsulitis OR rotator cuff OR (Shoulder AND (bursitis or frozen or impinge* or tendinitis or tendonitis or tendinopathy or pain)) AND (rehabilitat* or physiotherap* or physical therap* or manual therap* or mobili*) AND "spinal manipulative therapy" OR (manipulation AND (spinal OR spine OR thrust OR joint OR cervical OR thoracic OR shoulder OR shoulder girdle OR glenohumeral OR scapula OR scapulothoracic OR acromioclavicular OR musculoskeletal OR orthopedic)) OR (mobilization AND (spinal OR spine OR thrust OR joint OR cervical OR thoracic OR shoulder OR shoulder girdle OR glenohumeral OR scapula OR scapulothoracic OR acromioclavicular OR musculoskeletal OR orthopedic) OR (mobilisation AND (spinal OR spine OR thrust OR joint OR cervical OR thoracic OR Lumbar OR cervicothoracic OR shoulder OR shoulder girdle OR glenohumeral OR scapula OR scapulothoracic OR acromioclavicular OR musculoskeletal OR orthopedic) OR "chiropractic adjustment" OR ("mobilization with movement" OR "WM)) AND shoulder AND ((shoulder OR glenohumeral) AND (pain OR ache OR strain OR injur*)) OR shoulder girdle OR impingement OR rotator cuff OR acromioclavicular OR AC joint) AND adult AND Randomized Controlled Trial[pt] AND 2010/01/01:2023/08/03[dp]

MEDLINE

(randomized AND (control OR clinical)) AND shoulder pain OR shoulder impingement syndrome OR adhesive capsulitis OR rotator cuff OR (Shoulder AND (bursitis or frozen or impinge* or tendinitis or tendonitis or tendinopathy or pain)) AND (rehabilitat* or physiotherap* or physical therap* or manual therap* or mobili*) AND "spinal manipulative therapy" OR

(manipulation AND (spinal OR spine OR thrust OR joint OR cervical OR thoracic OR shoulder OR shoulder girdle OR glenohumeral OR scapula OR scapulothoracic OR acromioclavicular OR musculoskeletal OR orthopedic)) OR (mobilization AND (spinal OR spine OR thrust OR joint OR cervical OR thoracic OR shoulder OR shoulder girdle OR glenohumeral OR scapula OR scapulothoracic OR acromioclavicular OR musculoskeletal OR orthopedic) OR (mobilisation AND (spinal OR spine OR thrust OR joint OR cervical OR thoracic OR Lumbar OR cervicothoracic OR shoulder OR shoulder girdle OR glenohumeral OR scapula OR scapulothoracic OR acromioclavicular OR musculoskeletal OR orthopedic) OR "chiropractic adjustment" OR ("mobilization with movement" OR "WM)) AND shoulder AND ((shoulder OR glenohumeral) AND (pain OR ache OR strain OR injur*)) OR shoulder girdle OR impingement OR rotator cuff OR acromioclavicular OR AC joint) AND adult AND Randomized Controlled Trial[pt] AND 2010/01/01:2023/08/03[dp]

Limiters: peer-reviewed, full text, English language, Date range 2010-2023.

CENTRAL

AB((randomized AND (control OR clinical)) AND shoulder pain OR shoulder impingement syndrome OR adhesive capsulitis OR rotator cuff OR (Shoulder AND (bursitis or frozen or impinge* or tendinitis or tendonitis or tendinopathy or pain)) AND (rehabilitat* or physiotherap* or physical therap* or manual therap* or mobili*) AND "spinal manipulative therapy" OR (manipulation AND (spinal OR spine OR thrust OR joint OR cervical OR thoracic OR shoulder OR shoulder girdle OR glenohumeral OR scapula OR scapulothoracic OR acromioclavicular OR musculoskeletal OR orthopedic)) OR (mobilization AND (spinal OR spine OR thrust OR joint OR cervical OR thoracic OR shoulder OR shoulder girdle OR glenohumeral OR scapula OR

scapulothoracic OR acromioclavicular OR musculoskeletal OR orthopedic) OR (mobilisation AND (spinal OR spine OR thrust OR joint OR cervical OR thoracic OR Lumbar OR cervicothoracic OR shoulder OR shoulder girdle OR glenohumeral OR scapula OR scapulothoracic OR acromioclavicular OR musculoskeletal OR orthopedic) OR "chiropractic adjustment" OR ("mobilization with movement" OR "MWM")) AND shoulder AND ((shoulder OR glenohumeral) AND (pain OR ache OR strain OR injur*)) OR shoulder girdle OR impingement OR rotator cuff OR acromioclavicular OR AC joint) AND adult AND "Randomized Controlled Trial")

Custom Range: 2010-2023

CINAHL

AB((randomized AND (control OR clinical)) AND shoulder pain OR shoulder impingement syndrome OR adhesive capsulitis OR rotator cuff OR (Shoulder AND (bursitis or frozen or impinge* or tendinitis or tendonitis or tendinopathy or pain)) AND (rehabilitat* or physiotherap* or physical therap* or manual therap* or mobili*) AND "spinal manipulative therapy" OR (manipulation AND (spinal OR spine OR thrust OR joint OR cervical OR thoracic OR shoulder OR shoulder girdle OR glenohumeral OR scapula OR scapulothoracic OR acromioclavicular OR musculoskeletal OR orthopedic)) OR (mobilization AND (spinal OR spine OR thrust OR joint OR cervical OR thoracic OR shoulder OR shoulder girdle OR glenohumeral OR scapula OR scapulothoracic OR acromioclavicular OR musculoskeletal OR orthopedic) OR (mobilisation AND (spinal OR spine OR thrust OR joint OR cervical OR thoracic OR Lumbar OR cervicothoracic OR shoulder OR shoulder girdle OR glenohumeral OR scapula OR scapulothoracic OR acromioclavicular OR musculoskeletal OR orthopedic) OR "chiropractic

adjustment" OR ("mobilization with movement" OR "MWM")) AND shoulder AND ((shoulder OR glenohumeral) AND (pain OR ache OR strain OR injur*)) OR shoulder girdle OR impingement OR rotator cuff OR acromioclavicular OR AC joint) AND adult AND “Randomized Controlled Trial”)

Date range: January 2010- August 2023

Proquest

AB((randomized AND (control OR clinical)) AND shoulder pain OR shoulder impingement syndrome OR adhesive capsulitis OR rotator cuff OR (Shoulder AND (bursitis or frozen or impinge* or tendinitis or tendonitis or tendinopathy or pain)) AND (rehabilitat* or physiotherap* or physical therap* or manual therap* or mobili*) AND "spinal manipulative therapy" OR (manipulation AND (spinal OR spine OR thrust OR joint OR cervical OR thoracic OR shoulder OR shoulder girdle OR glenohumeral OR scapula OR scapulothoracic OR acromioclavicular OR musculoskeletal OR orthopedic)) OR (mobilization AND (spinal OR spine OR thrust OR joint OR cervical OR thoracic OR shoulder OR shoulder girdle OR glenohumeral OR scapula OR scapulothoracic OR acromioclavicular OR musculoskeletal OR orthopedic) OR (mobilisation AND (spinal OR spine OR thrust OR joint OR cervical OR thoracic OR Lumbar OR cervicothoracic OR shoulder OR shoulder girdle OR glenohumeral OR scapula OR scapulothoracic OR acromioclavicular OR musculoskeletal OR orthopedic) OR "chiropractic adjustment" OR ("mobilization with movement" OR "MWM")) AND shoulder AND ((shoulder OR glenohumeral) AND (pain OR ache OR strain OR injur*)) OR shoulder girdle OR

impingement OR rotator cuff OR acromioclavicular OR AC joint) AND adult AND "Randomized Controlled Trial")

Publication date: after 01 January 2010

PEDRO

Advanced Search:

Therapy: stretching, mobilization, manipulation, massage

Method: clinical trial

Body part: upper arm, shoulder, shoulder girdle

Published since: 2010

Score of at least: 6/10
